# Diagnosing Breastfeeding Difficulties: Where Do We Stand?

**DOI:** 10.1111/mcn.70153

**Published:** 2025-12-12

**Authors:** Laura Galante, Eriko Kobayashi, Miyu Nishikawa

**Affiliations:** ^1^ Swansea University Medical School Swansea UK; ^2^ Faculty of Nursing Toyama Prefectural University Toyama Japan; ^3^ Department of Biotechnology Faculty of Engineering Toyama Prefectural University Toyama Japan; ^4^ Division of Agriculture, Faculty of Agriculture University of Miyazaki Miyazaki Japan

**Keywords:** breastfeeding difficulties, breastfeeding support, human milk, impaired lactation, lactation biomarkers, milk biomarkers, secretory activation

## Abstract

Despite lactation being a natural occurrence in mammals, many structural barriers and individual factors can impact the ability of a woman to breastfeed her newborn. At the individual level, evidence has widely documented several risk factors and societal barriers for impaired lactation, many of which have been steeply increasing in human societies in the past few decades (e.g., psychosocial stress, metabolic disorders, births interventions, etc.). Yet the healthcare system worldwide does not seem to be prepared to support women facing such breastfeeding difficulties. Pregnant women are often provided with unrealistic expectations of how the breastfeeding experience should unfold, which can then translate into negative feelings when they encounter difficulties. In this context, the development of objective diagnostic tools able to help healthcare professionals and women identify breastfeeding difficulties that could then be treated accordingly would seem an ideal solution. Previous studies have tried to provide evidence for the use of milk compositional variations during early lactation as a tool to identify delayed secretory activation of the mammary gland, which often results in impaired lactation. However, despite portable technology for this purpose being successfully developed and/or validated, a consistent research gap remains around the true diagnostic power of such biomarkers in relation to clinically significant outcomes. This obstructs the development of effective diagnostic tools that could be employed in clinical practice to improve breastfeeding outcomes and breastfeeding rates.

## Summary

- Many risk factors for impaired lactation, including psychosocial stress, metabolic disorders and birth interventions are increasing in prevalence. Nonetheless lactation difficulties are not recognised and addressed in time in the clinical environment.
- Compositional changes of the milk have been investigated for decades as a possible diagnostic tool but a knowledge gap persists in relation to their usability and long‐term diagnostic power.
- To reduce this knowledge gap, future research should evaluate the usability of such tools and their diagnostic power with longitudinal study designs.
- Easily applicable diagnostic tools have the potential to improve the breastfeeding experience and outcomes for many women.

## 1 Background

Maternal milk is nature's response to the newborn's nutritional and physiological needs. Yet, despite the world observing steadily increasing breastfeeding rates (WHO UNICEF 2023) many women report the inability to produce enough milk for their child (Huang et al. 2022). As described by the three articles of the most recent Lancet Series on breastfeeding (Baker et al. 2023; Pérez‐Escamilla et al. 2023; Rollins et al. 2023), these worries are often inappropriately addressed by healthcare professionals through the introduction of supplemental feeds with ultra‐processed formula milk which in turn decrease maternal supply and the likelihood of ever returning to exclusive breastfeeding. As suggested by Farah et al. in a recent review, impaired lactation is not currently considered as a health issue per se (Farah et al. 2021). Prevalence rates are not defined, and diagnostic tools do not exist (Farah et al. 2021; Shere et al. 2021; Stuebe 2021). Yet while impaired lactation claims by women are often addressed as the result of inadequate breastfeeding practices, existing literature suggests that many risk factors for impaired lactation, including psychosocial stress, metabolic disorders and birth interventions are ever more widespread in our societies, generating a gap between breastfeeding women's needs and clinical knowledge and management of the condition. Additionally, compositional changes in the milk of women reporting lactation issues including mastitis have been documented (Boix‐Amorós et al. 2020; Liu et al. 2022; Mizuno et al. 2012), with possibly unknown effects on infant growth and development. A few authors to date have raised awareness regarding the importance to have appropriate diagnostic tools to identify women that might be struggling to attain or maintain appropriate milk volumes and with breastfeeding in general (Shere et al. 2021; Stuebe 2021). Hence, the present work aims at reviewing the existing evidence on such possible diagnostic tools and identifying possible gaps that are preventing their translation into practice.

## 2 Pathophysiology of Lactation

The production of milk through the mammary gland is a distinctive reproductive feature of mammalian species, and has evolved to sustain life while newborns are not able to feed otherwise (Oftedal 2012). The development, anatomy and physiology of the mammary gland, including during the lactation process, have been previously described by many authors (Anderson et al. 2007; Czank et al. 2007; Farah et al. 2021; McManaman and Neville 2003; Neville 2001, 2009b, 2009a; Neville et al. 1983; Neville and Neifert 1983; Pang and Hartmann 2007a, 2007b). These publications highlight that the lactation process can be clearly divided into two stages. The first stage starts during pregnancy, around the 24th week of gestation with the first production of small amounts of milk (i.e. secretory differentiation, previously known as lactogenesis I (Pang and Hartmann 2007a)). After birth the decline in the concentration of progesterone is the main trigger for the initiation of the second stage of lactation (i.e. secretory activation, previously known as lactogenesis II (Czank et al. 2007; Pang and Hartmann 2007a)). This usually occurs within 48–72 h from parturition (Pang and Hartmann 2007a). Hence the lactation process is established and maintained by a complex set of neuroendocrine mechanisms that after birth stimulate hormonal changes which activate a cascade of biological processes responsible for the end goal: milk production (Pang and Hartmann 2007a).

Notwithstanding the fact that lactation is a natural occurrence in mammals, a number of diverse structural barriers and individual‐level factors can and do impact on the ability of a woman to breastfeed or produce enough milk for their newborn. In their recent review Farah et al. identify and classify a series of individual level biomedical risk factors for impaired lactation (Farah et al. 2021). This list includes some major chronic conditions that are on the rise worldwide such as maternal metabolic conditions. This does not come as a surprise, as a growing amount of evidence shows that maternal BMI and metabolic disorders do indeed have a negative impact on breastfeeding outcomes and are significantly associated with the amount of milk produced by women (Nguyen et al. 2019; Segura‐Pérez et al. 2022; Suwaydi et al. 2022; Turcksin et al. 2014). Mechanisms of these associations have been investigated on animal models and suggest the involvement of disrupted hormonal pathways that are crucial for the initiation and continuation of lactation. Such evidence has been extensively reviewed by Luzardo‐Ocampo et al. in a recent publication (Luzardo‐Ocampo et al. 2023). Issues with the establishment of breastfeeding soon after birth is another important risk factor for impaired lactation, as the most important determinant for the maintenance of milk supply is the hormonal response generated by the suckling infant (Parker et al. 2015) and the effective removal of the milk produced. This mechanism generates a self‐sustaining “supply and demand” system which is easily affected by the inability of the newborn to draw milk efficiently or by the introduction of formula feeds which diminish the infant's need of maternal milk. As such a series of conditions including cleft lip/palate, ineffective or weak suck, ankyloglossia and lip tie can prevent the rapid establishment of breastfeeding after birth (Cregan et al. 2002; Farah et al. 2021). Moreover, evidence shows that infants whose mothers receive epidural anaesthesia, synthetic oxytocin and those who underwent assisted delivery or a traumatic birth experience were also less likely to successfully establish breastfeeding possibly due to a variety of causes including the disruption of natural suckling reflexes and maternal feeling of failure (Brimdyr et al. 2015; Marín Gabriel et al. 2015; Hongo et al. 2022; Omaru et al. 2024; Takahashi et al. 2021; Chatzopoulou et al. 2023). Receiving a large amount of intravenous fluid during labour (such as the induction drug *Syntocinon*® (Oxytocin | Drugs | BNF | NICE n.d.) are also negatively associated with breastfeeding (AIMS n.d.; Giudicelli et al. 2022). Issues in establishing milk supply soon after birth are also faced by most women who give birth prematurely, making preterm birth another risk factor for inadequate milk supply (52% of preterm women vs. 17% of term women) (Hill et al. 2005) Many factors associated with premature delivery can obstruct or delay the establishment of milk supply. These include the physical separation between mother and infant, the infant's immature oral reflexes, including arrhythmic suckling and poor ability to coordinate suck‐swallow‐breath patterns (Gewolb et al. 2001), and already mentioned pre‐existing conditions such as obesity or gestational diabetes, which are also risk factors for preterm delivery (Ye et al. 2022). Additionally, undergoing a caesarean section (Murase et al. 2014), and being exposed to antenatal glucocorticoids (Henderson et al. 2008) are also factors associated with a delay in the secretory activation of the mammary gland and low milk volumes. These factors too usually coincide with a preterm delivery. Exposure to stress, including psycho‐emotional stress is also a potential disruptor of normal lactation physiology (Nagel et al. 2022). Finally, genetic variations have been reported as a risk factor for low milk supply (Golan and Assaraf 2020) (Golan and Assaraf 2020), and these typically involve variations in prolactin or prolactin receptors which are also often related to cases of infertility, suggesting that mothers with history of infertility might be in need to be provided with additional support.

## 3 Challenges for the Healthcare System

**Figure 1:**
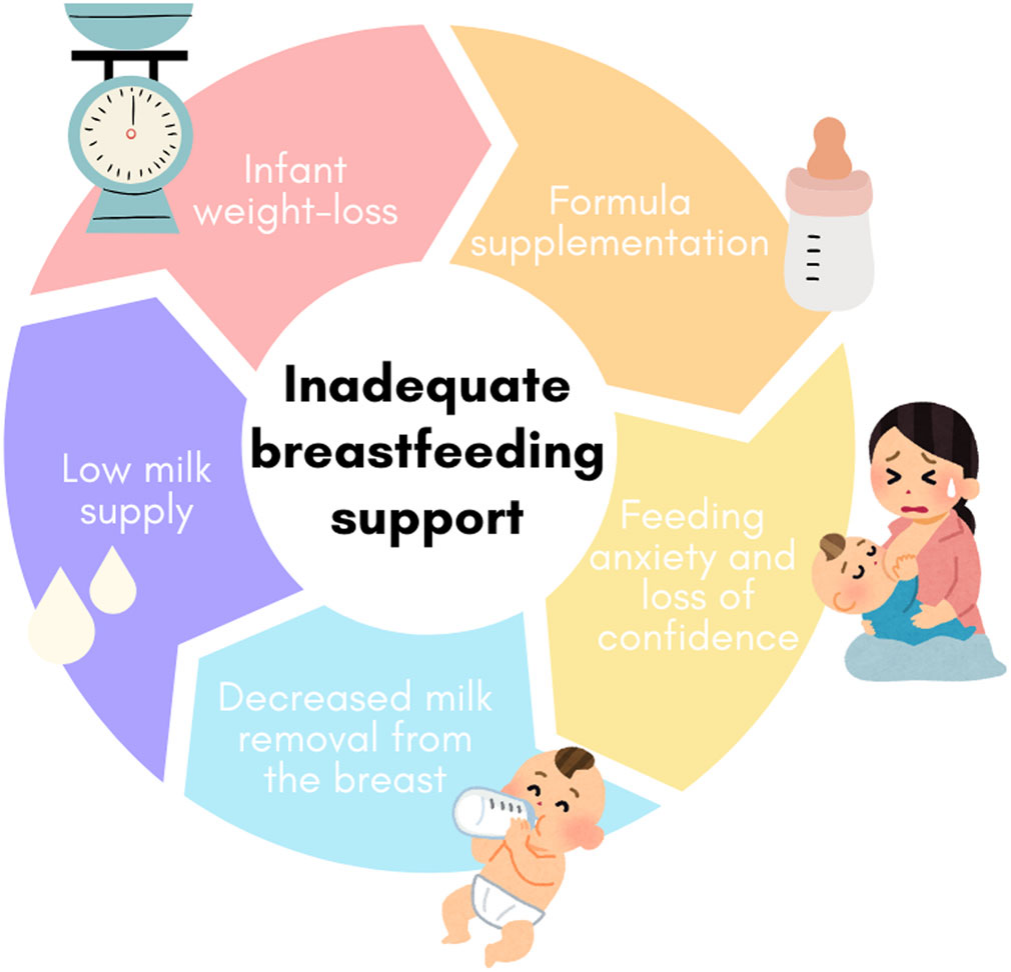
The vicious cycle of inadequate breastfeeding support.

Nonetheless, while existing evidence has widely documented several biomedical and non‐biomedical risk factors for impaired lactation, the healthcare system worldwide is currently not equipped to detect in time and timely address breastfeeding difficulties as a health problem that warrants diagnostic tools and a thorough preparation to support women who face it. Pregnant women are often provided with unrealistic expectations of the breastfeeding process (Fox et al. 2015; Giannì et al. 2020; Nardella et al. 2025) which increases negative feelings if the experience does not go accordingly. Previous qualitative studies on mothers' breastfeeding intentions and experiences show that willingness to breastfeed and being well‐informed are not always a solution to prevent early cessation of breastfeeding (Spannhake et al. 2021, 2022). Women who are willing to breastfeed and knowledgeable about its benefits but still face structural barriers manifested as individual level difficulties often encounter an inadequate healthcare response to their needs. This often comes in the form of perceived hostility and or misleading, conflicting and unclear information (Blixt et al. 2019; Spannhake et al. 2021, 2022). Misleading or unclear information provided by a healthcare professional that is expected to help women navigate one of the most delicate times in their lives is particularly worrying and stems from the current unpreparedness of the healthcare system to deal with breastfeeding difficulties as a real public health problem (Bahawi et al. 2023; Blixt et al. 2019; Esselmont et al. 2018; Shere et al. 2021). Figure 1 shows the vicious cycles created by the lack of timely breastfeeding support when mothers experience breastfeeding difficulties soon after giving birth. An important step towards finding a solution to this challenge might be the development of objective biomarker prognostic tools that can assist healthcare professionals and women in identifying, perhaps also predicting, and acknowledge issues with milk supply, and consequently refer women to high quality support that can help them identify the root cause of it and timely correct it. However, does such a tool exist and if not, can it be developed?

## 4 Diagnosing Breastfeeding Difficulties

Over the past few decades, researchers in the field of human lactation have attempted to develop diagnostic biomarkers based on our knowledge of lactation physiology. Indeed, the establishment of the mammary gland secretory activation is marked by a change in maternal metabolism but also in the metabolic profile of the milk itself, as described by a few authors already in the 1980s and 90s (Kulski and Hartmann 1981; Neville et al. 1991). Such changes (summarised in Table 1) which are due to the closure of the tight junctions between the lactocytes that secrete the milk, and their metabolic activation, underpin the concomitant rise of milk volume secreted (Neville 2023).

**Table 1:** Summary of literature describing the changes in milk composition during the secretory activation stage of lactation.

Author, year	Type of publication	Lactation stage	Compositional changes
Kulski and Hartmann (1981)^a^	Research study	Late pregnancy colostrum to milk	↓ K and Ca ↑ Proteins, Na, Cl, Mg
Neville et al. (1991)	Research study	2–4 days postpartum	↓ Protein, Na, Cl, pH ↑ Lactose, citrate, glucose, free phosphate, calcium
Allen et al. (1991)	Research study	1–6 months postpartum	↓ Protein, Na, K, Cl, bound Ca ↑ Lactose, glucose, pH, and free Ca.
Boss et al. (2018)	Narrative literature review	0–8 days postpartum	↓ Protein and Na ↑ Lactose and citrate
Neville (2023)	Narrative literature review	2–3 days postpartum	↓ Na and Cl ↑ Lactose

Abbreviations: ↑, increase; ↓, decrease; Na, sodium; K, potassium; Cl, chloride; Ca, calcium; Mg, magnesium.

a Authors only had access to the abstract.

These observations gave rise to the idea that the concentrations of these and other milk components during the first days postpartum could provide insight on whether the mammary gland undergoes a physiological secretory activation or not, therefore promoting them as biomarkers of lactation difficulties. Evidence of the usefulness of these biomarkers to identify the physiological onset of the secretory activation was already available in the 1990s (Humenick et al. 1998; Morton 1994). In this context the concentrations of a few different biomarkers, primarily sodium, potassium, lactose, citrate and total protein, have been linked to delayed secretory activation in women of preterm infants (Hoban et al. 2018, 2021), in women with diabetes (Arthur et al. 1989; Neubauer et al. 1993) and in women with elevated BMI (Medina Poeliniz et al. 2022)

A few studies have tried to validate the use of these biomarkers for translation into practice, particularly in relation to pump‐dependent women of preterm infants (Parker et al. 2024). Nonetheless, while the evidence suggests that the use of these molecules as biomarkers might constitute an effective tool for the identification of women at risk of impaired lactation, it is not clear whether altered concentrations during early lactation are linked to lower breastfeeding rates in the long term. This is due to the generalised lack of long‐term follow‐ups of the existing studies. Additionally, there is little or no evidence on whether such biomarkers can be used outside of early lactation as a form of screening, and on whether the collection methods used in the literature are indeed the most suitable for this purpose. The majority of the studies reviewed here collected sequential milk samples during early lactation and over the 24 h period. These conditions would likely be not suitable for a population‐based screening test, and might not be acceptable for most women. The willingness of women to be tested is a particularly important aspect to consider when discussing translation to practice. Previous research in women shows that “lack of time” and “having more important things to do” are often reported as reasons for not attending health screenings (Bennett et al. 2018; Shpendi et al. 2025). However, if a 24 h collection of human milk is not viable then what is the most suitable methodology to be applied to detect relevant and significant changes in the milk composition, that would be able to predict or identify lactation difficulties?

Furthermore, while milk compositional alterations have been linked to other underpinning issues that may impact breastfeeding rates at any time during the lactation period, such as mastitis (Ingman et al. 2014; Perrella et al. 2022), most of the existing studies linking lactation biomarkers with milk supply and or infant feeding (summarised in Table 2) primarily focus on alterations in early lactation. While this is the most common time when lactation issues originate, focussing solely on early lactation means neglecting the cohort of women that might face loss of milk supply at a later stage or that experience issues with re‐lactation further along their feeding journey.

**Table 2:** Summary of studies linking biomarkers of secretory activation with milk secretion and/or breastfeeding rates.

Authors, year, country	Sample	Sample collection	Biomarkers	Analysis method	Biomarkers normal range	Findings
Arthur et al. (1989) Australia^a^	38 healthy women, 6 T1D women	0 –10 days postpartum	Citrate, lactose, glucose	Unspecified in the abstract	Unspecified in the abstract	T1D linked to delayed normalisation of the biomarkers.
Neubauer et al. (1993) US	33 women IDD, 33 non‐IDD, 11 reference.	0–3 months	Lactose, glucose, total N	Lactose and glucose: model 27 industrial analyser Total N:‐ micro‐kjeldahl method	Not used	IDD linked to delayed lactogenesis (more likely to occur with poor metabolic control).
Jane A. Morton (1994) US	130 women	3–8 days postpartum. Hand‐expression from each breast at 8am. Milk refrigerated for 24 h.	Na	Ion specific electrode	Na ≤ 16 mmol/L	Breastfeeding at FU: 95% with normal Na vs 55% with high Na
Humenick et al. (1998) US	41 women	6 days postpartum. Hand‐expression from each breast at 8am. Milk refrigerated for 24 h.	Na	Ion specific electrode	Na ≤ 16 mmol/L	Breastfeeding at FU: 80% with normal Na vs 50% with high Na vs 22% with high risk to early breastfeeding cessation (according to questionnaire) and high Na.
Cregan et al. (2002b) Australia	22 women of preterm infants (31–35 weeks gestation), 16 healthy women of term infants	Day 5 postpartum. 24 h pooled milk and expression over the 24 h from individual	Na, protein, citrate, lactose	Protein: Bio‐Rad protein assay kit; citrate and lactose: Arthur et al. 1989 (inaccessible full text); Na: Corning 435 Flame Photometer	Citrate < 2.2 mM; lactose < 116.0 mM; Na > 24.0 mM; total protein > 18.6 g/l	82% of preterm women had ≥ 1 biomarker out of range (23% citrate; 36% lactose; 68% Na; 14% protein). The higher the number of biomarkers in range the higher the mean milk volume.
Murase et al. (2017) US	192 women	Day 7 postpartum	Na, K, Na:K	Flame photometry (Cole‐Parmer Dual‐Channel Flame Photometer, Vernon Hills, IL)	Na:K < 75th percentile among the analytic subset	High Na:K in 42% of women with a milk supply concern vs 21% of women without milk supply concern. Breastfeeding at FU: 83% with high Na:K vs 93% without.
Hoban et al. (2018) US	16 women of preterm infants (< 33 weeks gestation) admitted in NICU	1–14 days postpartum	Na, protein, citrate, lactose	Protein: Modified Bradford method (Bio‐Rad protein assay dye reagent concentrate); lactose and citrate: enzymatic spectrophotometric with HPLC; Na: (B‐722) ion selective electrode sensor pad (Horiba, Japan)	Na: 1.20–23.0 mM; total protein: 9.2–31.7 g/L; lactose: 100–223 mM; citrate: 2.24–6.40 mM	Number of normal biomarkers positively correlated with milk volumes on day 3 and 5. Coming to volume linked with 4 normal biomarkers on day 4.
Parker et al. (2021) US	69 women of preterm infants ( ≤ 32 weeks' gestation and < 1500 g)	milk collected at volume attainment ( ≥ 20 mL over two consecutive expressions)	Lactose, Na	Lactose: enzymatic measurement; Na: ion selective electrode (B‐722; HORIBA, Japan)	Na < 20 mM; lactose > 160 m	Women with normal lactose and sodium had more milk on Days 28 and 42, those with only normal lactose levels had more milk on day 42 and those with only normal sodium levels on day 28.
Galante et al. (2021) New Zealand	149 women preterm infants (32‐35 weeks gestation)	Day 5 postpartum ‐ 4 months corrected age. right breast whole expression between 10am and 12pm 2–3 h after previous feed/expression	Protein	Bicinchoninic acid assay (BCA)	Not used	Higher milk protein linked with more formula consumption by infants at FU
Medina Poeliniz et al. (2022) US*	39 PD women (N = 17 with pre‐pregancy BMI < 27, N = 22 with BMI ≥ 27)	1–14 days postpartum	Na, K, Na:K	Unspecified in the abstract	Unspecified in the abstract	Na decreased faster between day 1‐7 in BMI < 27 vs BMI ≥ 27
Perrella et al. (2022), Australia*	44 women of preterm infants (29–34 weeks gestation) With 6 cases of mastitis in total.	0–8 weeks postpartum. Multiple 24 h milk samples	Na, K, Na:K	Ion selective metres (Horiba, Japan)	Unspecified in the abstract	All cases of mastitis had high Na:K and 50% had reduced milk supply.
Yuan et al. (2023) China*	66 PD women of preterm infants. (I1: hospital‐grade breast pump used for day 1–14; I2, hospital‐grade breast pump used on day 1–5 and electric breast pump on day 6–14; C: electric breast pump used on day 1–14).	1–14 days postpartum	Na	Unspecified in the abstract	Unspecified in the abstract	Normal Na on day 5: 73% of I1 and I2 vs 41% of C.

Abbreviations: Na, sodium; K, potassium; Na:K, sodium‐to‐potassium ratio; FU, follow‐up; T1D, type 1 diabetes; IDD, insulin dependent diabetes; PD, pump dependent; I, intervention; C, control.

* Authors only had access to the abstract.

Finally, the reliability of such biomarkers to predict the development of clinically significant alterations in the physiology of the mammary gland and, possibly, in the amount of milk produced, is still uncertain, as suggested by Pace et al. in a recent study on the use of sodium and sodium‐to‐potassium ratio in the context of clinical mastitis (Pace et al. 2022).

On the other hand, very promising findings come from studies that have validated and/or piloted the use of portable devices for the measurement of some of these biomarkers (i.e. sodium and potassium) (Esquerra‐Zwiers et al. 2022; Furukawa et al. 2022; Haramati et al. 2023; Lai et al. 2018), providing quick, easy and affordable methodology that can be used in primary healthcare settings and or at home by women. In this context promising interventions are also starting to be developed based on the use of these biomarkers (e.g a biomarker‐based texting app based on sodium measurement (Magalhães et al. 2023)).

Yet, the considerable gaps in our understanding of the diagnostic power of these biomarkers, together with the inconsistency in the collection and analytical methodologies used in the existing studies, make it hard to harness a consensus on whether these biomarkers can be translated into practice and applied to provide effective support to healthcare professionals, women and infants.

## 5 Conclusions

Overall, the evidence reviewed here suggests that while well‐established pathways in the physiology of the lactating breast have been studied and proposed as a means to identify potential issues with lactation and milk supply, the diagnostic power of these biomarkers has not been thoroughly tested against longer term clinical outcomes such as infant weight‐gain and the timely reaching of developmental milestones, as well as in association to the introduction of formula supplementation and the cessation of breastfeeding. Additionally, in light of the variability of milk composition more research should be conducted on whether different methods of collection reflect on the variability of the biomarkers and on their diagnostic power. As such, we suggest that future research in the following areas is particularly important in relation to the development of an accessible and sustainable diagnostic tool:

- The relationship between sample collection methodology and diagnostic power of various biomarkers.
- The predictive power of various biomarkers in relation to early cessation of breastfeeding.
- The diagnostic power of various biomarkers at different times during lactation.
- The feasibility of a standardised collection methodology for population‐based screening.
- The relationship between receiving a diagnosis vs not receiving a diagnosis in relation to long‐term breastfeeding outcomes and quality of the breastfeeding experience.

Addressing such gaps will ensure that the efforts spent, and the tools developed to equip the healthcare system in assisting nursing women, can be both effective and sustainable.

Yet, and most importantly, given the multifactorial origin of lactation difficulties a thorough investigation of the topic warrants a multidisciplinary approach and research agenda where the multiple societal, clinical and biological risk factors are taken into consideration. Only this will enable us to improve breastfeeding interventions and empower women to attain their breastfeeding goals, rather than “normalising” the inability to reach them, which can come with a very heavy load on their mental health (Rowles et al. 2025).

## Author Contributions

All authors conceptualised the manuscript. L.G. wrote the first draft. E.K. retrieved some of the literature. L.G., E.K. and M.Y. reviewed and approved the final version of the manuscript.

## Conflicts of Interest

The authors declare no conflicts of interest.

## Data Availability

Data sharing not applicable to this article as no datasets were generated or analysed during the current study.
